# Dynamic associations of neighborhood social and physical environment with ED visits for asthma: A spatial change score analysis

**DOI:** 10.1016/j.jacig.2026.100672

**Published:** 2026-02-16

**Authors:** Yonsu Kim, John Cho, Ji Won Yoo, Chris Cochran, Sheniz Moonie

**Affiliations:** a Department of Healthcare Administration and Policy, School of Public Health, University of Nevada, Las Vegas, Nev; d Department of Epidemiology and Biostatistics, School of Public Health, University of Nevada, Las Vegas, Nev; c Department of Internal Medicine, Kirk Kerkorian School of Medicine, University of Nevada, Las Vegas, Nev; b Southern California Association of Governments, Los Angeles, Calif

**Keywords:** Asthma, ED visits, built environment, asthma disparities, neighborhood environment, disadvantaged neighborhoods, PM_2.5_, spatial regression

## Abstract

**Background:**

A growing number of studies identified risk factors for asthma in neighborhood social and environmental contexts. Yet, there is a lack of comprehensive knowledge about the time-varying relationship between neighborhood environmental and socioeconomic factors and asthma outcomes.

**Objectives:**

This study aimed to assess the variations in neighborhood environmental, socioeconomic, and demographic characteristics; to investigate the census tracts in Los Angeles County where emergency department (ED) visits for asthma increased; and to identify neighborhood characteristics whose change contributed to alleviating or exacerbating the burden of asthma in health care use.

**Methods:**

Using an ecological, retrospective study design, we applied a change score method, spatial regression and geographic information system analysis to examine time-varying effects of neighborhood characteristics in Los Angeles County.

**Results:**

The average ED visits for asthma showed modest increases. Despite of overall improvements in neighborhood socioeconomic status, no beneficial impact of neighborhood socioeconomic status was found except median household income. Increased median household income was associated with a decrease in ED visits in socioeconomic domain. Increased particulate matter with diameter <2.5 μm (PM_2.5_) was associated with an increase in ED visits for asthma in environmental domain. The positive association between PM_2.5_ and ED visits for asthma was validated by the spatial pattern analysis.

**Conclusions:**

This study identified PM_2.5_ as a neighborhood environmental risk factor for asthma. However, little evidence was found for the beneficial effect of improved neighborhood socioeconomic status. Our findings underscore that, in the short term, reducing traffic emissions may contribute more to lowering the burden of asthma-related health care use than improving neighborhoods’ socioeconomic status.

Asthma is one of the chronic diseases that is significantly influenced by the neighborhood environment, along with cardiovascular disease, obesity, type 2 diabetes, and mental health disorders. A growing number of studies examined how neighborhood characteristics were associated with incidence, morbidity, and health care use for asthma and addressed risk factors in the neighborhood social and environmental contexts.^1^, ^2^, ^3^, ^4^, ^5^ These neighborhood characteristics play a key role in explaining the higher rates of asthma exacerbations and hospital-based care use for asthma in disadvantaged neighborhoods. The recent studies have focused more extensively on ecological factors rather than biological or individual-level factors, particularly to uncover underlying causes of asthma disparities in urban areas.^6^, ^7^, ^8^

Neighborhood environmental characteristics include air pollutants, transportation-related factors, land use, and density. Among these, air pollutants have been identified as among the key risk factors contributing to the onset and development of asthma in epidemiologic studies. A meta-analysis reported that exposure to particulate matter <2.5 μm (PM_2.5_), PM <10 μm (PM_10_), Nitrogen Dioxide (NO2), and black carbon was associated with the risk of asthma incidence and development.^9^ Increased exposure to PM2.5, nitrogen oxides, and CO was associated with increased risk of asthma diagnosis, while decreased exposure to PM_2.5_ and NO_2_ was associated with a decreased incidence of asthma among children.^7^

In particular, CO, nitrogen oxides, and PM are released into the atmosphere from vehicle fuel combustion. Thus, traffic conditions should be closely linked to asthma symptoms through traffic emissions. Empirical studies assessed various built environment characteristics that may affect traffic emissions in urban areas. Features such as lower traffic density, higher residential density, mixed land use, and greater walkability were found to reduce traffic volume and vehicle emissions. This reduction, in turn, leads to a reduction in asthma symptoms.^10^ Kim et al^11^ found that an increased share of land use mix (LUM) and open/recreation land was associated with a reduction in emergency department (ED) visits for asthma.

Neighborhood socioeconomic status (SES) is characterized by household income, educational attainment, employment, and access to resources at the neighborhood level.^12^ These factors are also associated with higher prevalence and incidence of asthma among marginalized populations, leading to racial/ethnic disparities in asthma outcomes. Adverse asthma outcomes are more pronounced in disadvantaged neighborhoods, which are often defined by higher poverty rates, limited educational attainment, and lower household income.

More specifically, the rates of asthma exacerbations and ED visits are higher among the neighborhoods with a high poverty rate, which tend to struggle with lower educational attainment and higher unemployment rates. Along with individual-level socioeconomic determinants, living in low-income, high-poverty neighborhoods is associated with increased asthma prevalence and exacerbation, particularly among marginalized racial and ethnic groups.^11^^,^^13^^,^^14^

Neighborhood SES is closely linked to demographic factors. Asthma prevalence and hospital-based care for asthma were significantly higher in those neighborhoods with higher proportions of African American and Hispanic populations in Los Angeles County (LAC).^11^^,^^15^ Environmental and socioeconomic factors may disproportionately exacerbate asthma symptoms among marginalized populations residing in disadvantaged communities.

Previous studies provided strong evidence for neighborhood environmental and socioeconomic risk factors for asthma. However, most have been restricted to cross-sectional data capturing these characteristics at only a single point in time, overlooking time-varying effects. Furthermore, they provided limited evidence for temporal trends in neighborhood risk factors and the mechanism by which those neighborhood risk factors contribute to asthma exacerbation and increased health care use. One study examined how temporal changes in air pollutants contributed to a reduction in childhood asthma at the individual level.^16^ Yet, there is a lack of comprehensive knowledge about the time-varying relationship between neighborhood context and asthma outcomes at the neighborhood level.

To fill this gap, this study aimed to measure the variations in neighborhood characteristics across the 3 domains, to identify the census tracts where ED visits for asthma increased in LAC, and to examine neighborhood characteristics whose change contributed to either alleviating or exacerbating the burden of asthma in health care use.

## Methods

### Data

We retrieved the age-adjusted rate of ED visits for asthma from CalEnviroScreen (CES) versions 3.0 and 4.0 (California Office of Environmental Health Hazard Assessment [OEHHA]; https://oehha.ca.gov/calenviroscreen/report/calenviroscreen-40) as the outcome variable. CES is a screening tool designed to assess the burden of pollution in California neighborhoods. It provides data on exposures, socioeconomic factors, and sensitive populations, along with asthma ED visits at the census tract level. CES 3.0 was released in 2017, and CES 4.0 was released in 2021. Both provide datasets of PM_2.5_ (annual mean PM_2.5_ concentrations), diesel PM (diesel PM emissions from on-road and nonroad sources), traffic density (traffic density, in vehicle kilometers per hour per road length, within 150 meters of the census tract boundary), education (percentage of population over age 25 with less than a high school education), poverty (percentage of population living below 2 times the federal poverty level), and unemployment (percentage of the population over age 16 that is unemployed and eligible for the labor force).

We also obtained data on employment density, street density, LUM (the degree of diversity in land uses) index, and percentage of open space from Southern California Association of Governments. Additional neighborhood indicators were retrieved from the American Community Survey, such as percentage of “less than high school,” median household income ([MHI], the middle-income level at which one-half of households earn more and one-half earn less), percentage of “below the federal poverty level,” percentage of children, percentage of African Americans, household size, and year structure built for the corresponding years. After combining those variables from the 3 sources, we selected 2,334 census tracts without missing values from 2,496 census tracts in LAC, which had a population of approximately 10 million in 2020, making it as the nation’s most populous county.

Referring to the previous studies,^5^^,^^17^ we categorized neighborhood characteristics into 3 domains, including (1) environmental (density measures, air pollution emissions, traffic conditions, and LUM), (2) socioeconomic (percentage of” less than high school,” percentage of “below the federal poverty level,” unemployment rate, MHI, and household size), and (3) demographic (racial/ethnic decomposition, percentage of children under age 10) domains. This study was exempt from Institutional Review Board review as it used publicly available health outcome data at the aggregate level.

### Statistical analyses

We employed an ecological, retrospective study design, and a change score analysis that evaluates time-varying effects of neighborhood characteristics at 2 time points and provides a framework for causal inference. To apply this method, we calculated the difference (ie, Δ) in each neighborhood variable as well as ED visits between the 2 time points. These differences were used as independent and dependent variables to examine whether changes in neighborhood characteristics were associated with changes in ED visits for asthma at the census tract level in LAC.

For statistical analysis, we first used the linear regression (ordinary least square [OLS]) analysis. Then, we assessed the presence of autocorrelation using Moran’s *I* and applied the spatial autoregressive model ([SAR], a spatial regression model that accounts for spatial dependence among the dependent variables). SAR is commonly used when spatial dependence in the dependent variable exists in spatial data. Such spatial dependence may occur when an outcome in a location affects nearby locations (eg, spillover). For spatial lag in the SAR equation, spatial weights matrix indicates nearby locations and measures weight (*W*) each neighbor contributes to the dependent variable. SAR coefficient (*ρ*) indicates how strongly outcomes in nearby census tracts affect the outcome in a given census tract. We presumed that the residuals from the OLS might exhibit spatial autocorrelation in LAC spatial data and applied the spatial error model ([SEM], a spatial regression model that accounts for spatial dependence in the error terms). After running SAR and SEM for all census tract data (ALL), we repeated the analyses separately for the census tracts with an increase in ED visits (RISE) and those with a decrease (DROP).

$$\Delta y=\rho W\Delta y+\Delta X_{1}\beta_{1}+\Delta X_{2}\beta_{2}+\Delta X_{3}\beta 3+\varepsilon$$

where Δy = vector of changes in ED visits for asthma; ρWΔy = spatial lag of change in ED visits for asthma; $\Delta X_{1}$ = matrix of changes in demographic variables; $\Delta X_{2}$ = matrix of changes in environmental variables; $\Delta X_{3}$ = matrix of changes in socioeconomic variables; $\beta_{1}\beta_{2}\;\beta_{3}$ = parameters for $\Delta X_{1}$*,* $\Delta X_{2}$*,* $\Delta X_{3}$*; ε* = vector of error term.

Geographic information system mapping was used to visualize the spatial patterns of ED use for asthma and demographic, socioeconomic, and environmental variables; identify patterns that might not be observed in statistical analysis; and detect spatial dependence. Local Moran’s *I* was used to identify hot spots of increased ED visits for asthma. This approach also allowed us to verify the associations that were assessed by statistical analysis. GeoDA was used for OLS, SAR, and SEM; and ArcGIS Pro was used for mapping.

## Results

### Descriptive statistics

The average ED visits for asthma increased by 2.2 per 100,000 population (SD = 9.9) between the 2 time points, ranging from −37.8 to 87.9. It increased among 1,393 census tracts (RISE: 59.3%) and decreased among 941 census tracts (DROP: 40.7%) (Table I). Air quality and environmental conditions showed mixed results. PM_2.5_ increased by 0.1 (SD = 0.7), ranging from −4.6 to 4.2. The increase in PM_2.5_ was detected in 1,228 census tracts, indicating a general deterioration of air quality across LAC. Unlike PM_2.5_, diesel PM showed a significant reduction. It fell in the most census tracts, indicating a decrease of 24.2 on average. Traffic density significantly rose by 130.6, showing an increase in 1,395 census tracts. In contrast, street density decreased slightly. LUM also showed a mild decrease by 0.1, whereas open space barely increased.

**Table I:** Summary statistics of changes in neighborhood characteristics for all census tracts (ALL), census tracts with increases in ED visits for asthma (RISE), census tracts with decreases in ED visits for asthma (DROP) in LAC

Variables	ALL	Minimum	Maximum	RISE	Minimum	Maximum	DROP	Minimum	Maximum
	Mean (SD)			Mean (SD)			Mean (SD)		
Asthma	2.2 (9.9)	−37.8	87.9	7.6 (8.4)	0.0	87.9	−5.8 (5.8)	−37.8	0.0
PM_2.5_	0.1 (0.7)	−4.6	4.2	0.2 (0.7)	−9.3	4.2	0.0 (0.6)	−4.6	3.6
Diesel PM	−24.2 (17.6)	−208.3	−0.1	−23.7 (15.4)	−208.3	−0.3	−24.9 (20.5)	−208.3	−0.1
HH size	0.0 (0.3)	−1.5	1.2	0.0 (0.3)	−1.3	1.2	0.0 (0.3)	−1.5	0.9
Traffic density	130.6 (477.3)	−1,700.8	4,308.4	110.3 (480.8)	−1,700.8	2,824.2	160.5 (470.7)	−1677.4	4,308.3
Emp density	−8.8 (411.9)	−19,876.2	331.7	−13.9 (532.6)	−19,876.2	331.7	−1.1 (42.4)	−19.8	21.6
Street density	0.0 (0.0)	−0.1	0.0	0.0 (0.0)	−0.1	0.0	−0.0 (0.0)	−0.1	0.1
LUM	−0.1 (0.1)	−0.7	0.5	−0.1 (0.1)	−0.7	0.5	−0.1 (0.1)	−0.6	0.5
Open, % Space	0.0 (0.1)	−0.2	0.8	0.0 (0.1)	−0.2	0.8	0.0 (0.1)	−0.1	0.8
Year built	0.1 (4.6)	−38.0	41.0	0.2 (4.8)	−38.0	41.0	0.0 (4.4)	−29.0	25.0
Education	−2.8 (5.9)	−29.7	19.0	−2.9 (6.1)	−29.7	19.0	−2.5 (5.4)	−21.8	13.4
Unemployment	−5.1 (4.7)	−25.6	21.6	−5.3 (4.7)	−25.6	12.0	−4.7 (4.6)	−19.8	21.6
MHI ($1K)	5.4 (11.0)	−89.6	87.3	5.0 (11.0)	−89.6	81.0	6.1 (11.3)	−38.2	87.3
Poverty, %	−6.1 (9.0)	−63.4	26.2	−6.2 (9.3)	−63.4	26.2	−6.1 (8.5)	−46.0	20.5
Pop density	112.2 (449.0)	−2,720.0	5,630.0	125.3 (471.3)	−2,720.0	5,630.0	93.1 (413.5)	−1,437.0	3,000.0
Whites, %	−1.6 (11.0)	−100.0	43.6	−1.5 (11.3)	−100.0	43.6	−1.8 (10.5)	−100.0	34.5
African Americans, %	−2.0 (15.7)	−22.9	22.4	−0.5 (3.8)	−22.9	22.4	−0.1 (3.6)	−20.4	22.4
Hispanics, %	0.5 (6.3)	−50.0	51.5	0.7 (6.2)	−19.4	51.5	0.2 (6.4)	−50.0	40.0
Asians, %	0.6 (4.1)	−32.0	21.7	0.6 (4.0)	−31.5	21.3	0.7 (4.3)	−17.5	21.7
n	2,334			1,393			941		

Note: Positive numbers indicate increases and negative numbers indicate decreases. Year built refers to year the structure was built.

*Emp,* Employment; *HH,* household; *Pop,* population.

The indicators in socioeconomic domain exhibited overall improvement on average. Percentage of “less than high school” declined by 2.8%. Unemployment rate also decreased by 5.1% and MHI increased by $5,439.70. Poverty rate decreased by 6.1%. Population density increased by 112.2 persons per square mile. Racial composition showed a secular pattern of population changes in LAC. Percentage of African American and White residents decreased overall by 2.0% and 1.6%, respectively, whereas percentage of Asians and Hispanics increased by 0.6% and 0.5%.

### Results of the statistical analysis

The results of OLS for the full model indicate that increases in PM_2.5_ (*β* = 3.14) and population density (*β* = 0.001) were associated with an increase in ED visits for asthma. However, increases in traffic density (*β* = −0.002), street density (*β* = −82.97), percentage of “less than high school” (*β* = −0.08), unemployment rate (*β* = −0.10), MHI (*β* = −0.1), and percentage of African Americans (*β* = −0.19) were negatively associated with an increase in ED visits for asthma.

Moran’s *I* confirmed the presence of spatial autocorrelation (ALL: 0.48; RISE: 0.55; DROP: 0.34). These values indicate moderate to strong spatial clustering of the dependent variable, supporting the use of spatial regression for this study. Thus, we applied SAR and SEM, using the original and *z*-score standardized variables. Queen contiguity was used to define the spatial weight matrix, which accounts for neighboring tracts that share either a boundary or a vertex. Based on the results of diagnostic statistics, we selected SAR as the best model, given the stronger robust LM (Lagrange Multiplier)-lag statistics (ALL: *P* < .001; RISE: *P* < .001, DROP: *P* < .01) than LM-error (ALL: *P* < .05, RISE: *P* = .15, DROP: *P* = .99) and lower Akaike information criterion statistics in SAR (ALL: 13,637, RISE: 7,848, DROP: 4,753) than in SEM (ALL: 13,645, RISE: 7,866, DROP: 4,758) across the 3 models (Table II). We performed the variance inflation factor tests and found no significant multicollinearity issues.

**Table II:** Results of SAR and SEM models: estimated *β*-coefficients for ALL, RISE, and DROP

Variable	ALL	*z*-score	SEM (*β*)	*z*-score	RISE	*z*-score	SEM (*β*)	*z*-score	DROP	*z*-score	SEM (*β*)	*z*-score
	SAR (*β*)				SAR (*β*)				SAR (*β*)			
	Original		Original		Original		Original		Original		Original	
*Ρ*	.66∗∗∗	.66∗∗∗			.60∗∗∗	.61∗∗∗			.37∗∗∗	.36∗∗∗		
λ			0.68∗∗∗	0.68∗∗∗			0.62∗∗∗	0.62∗∗∗			0.37∗∗∗	0.37∗∗∗
PM_2.5_	1.06∗∗∗	0.07∗∗∗	2.20∗∗∗	0.16∗∗∗	0.53∗∗∗	0.04∗∗∗	1.25∗	0.09∗	0.30	0.02	0.40	0.03
Diesel PM	0.00	0.02	0.02	0.03	0.00	0.00	−0.02	−0.03	0.02∗	0.04∗	0.03∗	0.06∗
HH size	0.16	0.004	0.10	0.00	0.37	0.01	0.35	0.01	−0.54	−0.01	−0.74	−0.02
Traffic density	−0.001	−0.001	0.00	0.01	0.00	−0.01	0.00	−0.01	0.00	0.02	0.00	0.03
Employment	−0.16	−6.57	−0.41	−17.08	0.22	8.99	0.07	2.92	−0.53	−22.07	−0.61+	−25.43+
Street density	−54.21∗	−0.04∗	−58.91∗	−0.04∗	−28.88	−0.02	−25.83	−0.02	−26.47	−0.02	−26.11	−0.02
LUM	−2.44	−0.02	−2.14	−0.02	−0.69	2.48	−1.25	−0.01	0.60	0.01	0.48	0.00
Open space, %	−0.21	0.00	1.07	0.01	−4.08	−0.01	−3.47	−0.02	6.08+	0.04+	6.03+	0.04+
Year built	−0.02	−0.01	−0.01	−0.01	−0.01	−0.01	0.01	0.01	−0.03	−0.01	−0.03	−0.01
Education	−0.04	−0.03	−0.03	−0.02	−0.02	−0.01	−0.01	−0.01	0.05	0.03	0.05	0.03
Unemployment	−0.02	−0.01	0.01	0.00	−0.03	−0.02	−0.01	−0.01	0.02	0.01	0.01	0.00
MHI ($1K)	−0.04∗	−0.04∗	−0.02	−0.02	−0.04∗	−0.05∗	−0.03	−0.03	0.00	0.00	0.00	0.00
Poverty, %	−0.02	−0.02	−0.02	−0.01	−0.05∗	−0.04∗	−0.04+	−0.03+	−0.02	−0.02	−0.02	−0.02
Pop density	0.00+	0.03+	0.00	0.02	0.001∗	0.04∗	0.00	0.02	0.00	−0.02	0.00	−0.02
Hispanics, %	0.01	0.01	0.01	0.01	0.05	0.04	0.04	0.02	−0.04	−0.03	−0.04	−0.02
African Americans, %	−0.17∗∗	−0.06∗∗	−0.17∗∗∗	−0.06∗∗∗	−0.11∗	−0.04∗	−0.10+	−0.04+	−0.02	−0.01	−0.02	−0.01

∗∗∗*P* < .001, ∗∗*P* < .01, ∗*P* < .05, +*P* < .10. Original refers to the *β*-coefficients using the original measurement units. *z*-score refers to the *β*-coefficients using *z*-score standardized variables. *ρ* refers to spatial autoregressive coefficient. *λ* refers to spatial error coefficient.

The results of SAR were generally consistent with those of OLS, with some nuanced differences. An increase in PM_2.5_ was associated with an increase in asthma ED visits in ALL (*β* = 1.06; *P* < .001) at a 95% confidence level. In contrast, a negative association was found for street density (*β* = −54.21; *P* < .05), MHI (*β* = −0.04; *P* < .05), and percentage of African Americans (*β* = −0.17; *P* < .01).

The results of RISE model appeared to be somewhat consistent with those of ALL, confirming the detrimental impact of increases in PM_2.5_ (*β* = 0.53; *P* < .001) and the beneficial impacts of increases in MHI (*β* = −0.04; *P* < .05) and percentage of African Americans (*β* = −0.11; *P* < .05). In the DROP model, however, only an increase in diesel PM was associated with increased ED visits (*β* = 0.02; *P* < .05).

The results of SAR in ALL suggest that there was an additional ED visit for asthma per 100,000 population for a 1-μg/m^3^ increase in PM_2.5_ concentration. Given the population of LAC, a 1-unit reduction in PM_2.5_ concentration could prevent approximately 100 asthma-related ED visits between the 2 time points. Relatively, there was a limited impact of household income on ED visits, although it is statistically significant. ED visits for asthma per 100,000 population decreased slightly by 0.04 for every $1,000 increase in MHI. The *z*-score standardized test was used to compare the effect sizes across the significant variable. The results indicated the highest standardized coefficient for PM_2.5_ (*β* = 0.07), suggesting a 1-SD increase in PM_2.5_ was associated with a 0.07-SD increase in ED visits.

### Results of spatial analysis

**Fig 1:**
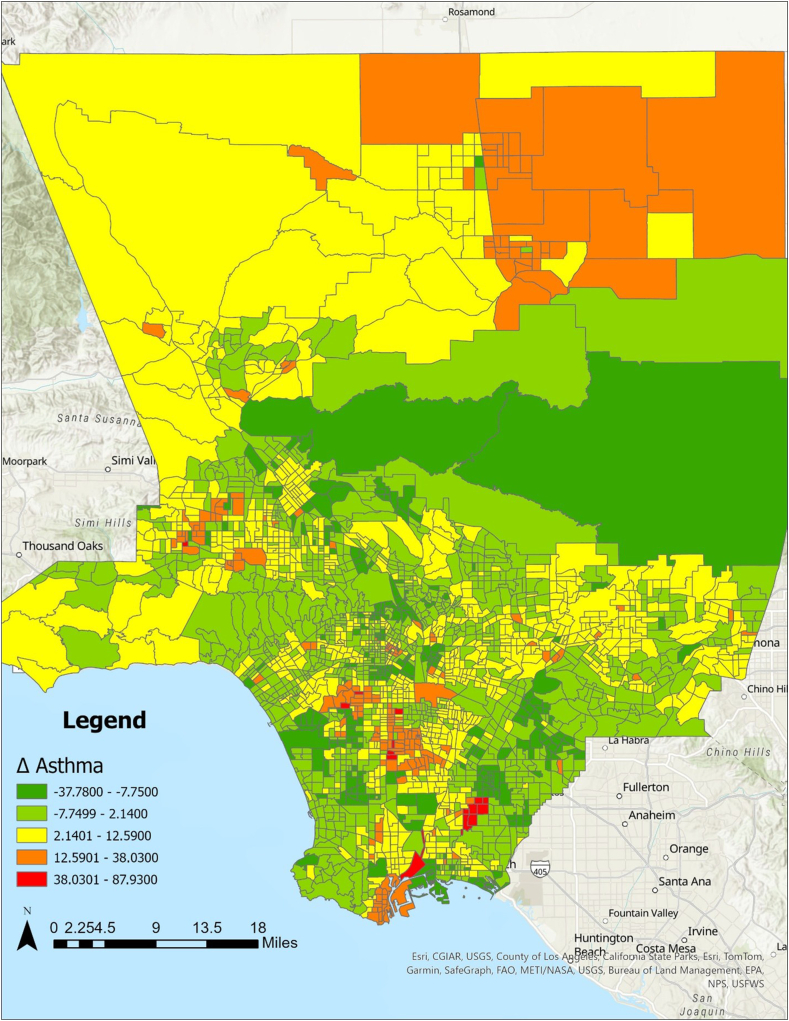
Difference (Δ) in asthma-related ED visits between CES3.0 and CES4.0 by census tract in LAC. Census tracts in *red* and *orange* indicate a significant increase in ED visits, while *green* indicates a decrease.

**Fig 2:**
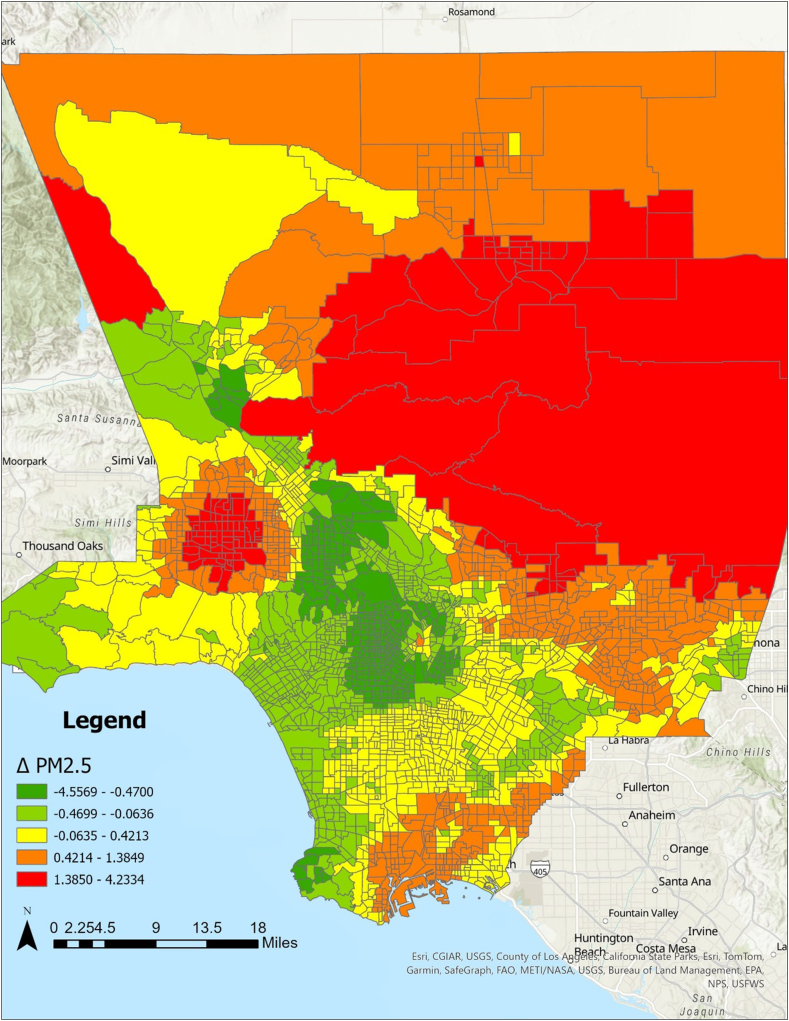
Difference (Δ) in PM_2.5_ between CES3.0 and CES4.0 by census tract in LAC. Census tracts in *red* and *orange* indicate an increase in PM_2.5_, while *green* indicates a decrease.

**Fig 3:**
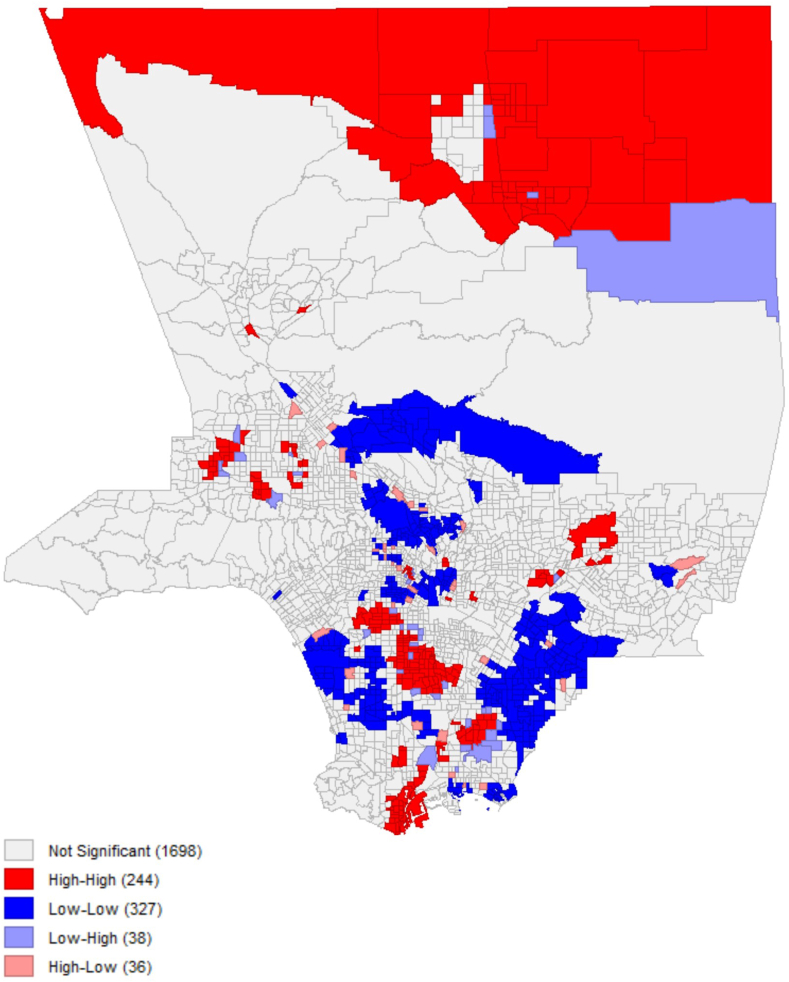
Spatial cluster of census tracts showing substantial variation in ED visits for asthma between CES3.0 and CES4.0. Census tracts in *red* indicate hot spots (high-high: areas with high values surrounded by high-value neighbors), while census tracts in *blue* indicate cold spots (low-low: areas with low values surrounded by low-value neighbors).

Fig 1 shows the spatial distribution of neighborhoods with a relatively larger increase in ED visits. Consistent with a recent cross-sectional study,^11^ a higher growth rate of ED visits was found in the neighborhoods located in Southern South Los Angeles, as well as West San Fernando Valley, and Southeast LAC. Mapping confirmed the strong spatial relationship between the key air pollutants and ED visits. The change in PM_2.5_ closely mirrored the spatial pattern of changes in ED visits, particularly in South Los Angeles, East San Gabriel Valley, and West San Fernando Valley (Fig 2). In particular, South Los Angeles was identified as a hot spot (high-high: areas with high values surrounded by high-value neighbors) for ED visits, aligning with literature (Fig 3).^11^ Conversely, little variation in PM_2.5_ was observed in central Los Angeles, including downtown, Koreatown, Mid-Wilshire, and Northern South Los Angeles. Although MHI was statistically significant in statistical analyses, we found no distinct geographic pattern of MHI. Likewise, mapping revealed no geographic patterns for other neighborhood variables.

## Discussion

This study examined the dynamic impact of neighborhood characteristics in LAC and found that an increase in PM_2.5_ was significantly associated with increased ED visits for asthma. In environmental domain, PM_2.5_ substantially increased across all census tracts in LAC between the 2 time points, and its increase was associated with the increase in ED visits for asthma. In socioeconomic domain, an increase in MHI was associated with a decrease in ED visits for asthma, while overall household income increased in LAC. In the spatial analyses, we found that a higher growth in ED visits was identified in some disadvantaged neighborhoods. These areas showed a relatively high rate of ED visits for asthma, suffering from high exposure to economic deprivation, lower educational attainment, poor air quality, and substandard housing conditions.

Among the traffic-related air pollutants, PM_2.5_ has emerged as a key risk factor for asthma in numerous studies. The literature reported the association between PM_2.5_ and asthma outcomes, such as ED visits, incidence, and onset and development of asthma. In particular, children were found to be more susceptible to long-term exposure to PM_2.5_.^18^, ^19^, ^20^ While the majority of studies relied on cross-sectional designs to examine the association between PM_2.5_ and asthma outcomes, few studies evaluated the dynamic impact of PM_2.5_ on ED visits (ie, to what extent ED visits for asthma change by a change in PM_2.5_). A longitudinal study assessed the impact of ambient air pollutants collected from monitoring stations in Southern California and found that decreases in PM_2.5_ and NO_2_ were associated with a reduction in asthma incidence among children.^16^ This study provided solid support for the findings of Garcia et al^16^ by extensively using neighborhood SES as well as environmental characteristics at the neighborhood level.

We found no compelling evidence for the association between neighborhood SES and ED visits. The increased MHI appeared to contribute to a reduction in ED visits for asthma, whereas poverty rate, educational attainment, and unemployment rate showed mixed or insignificant results. Several studies demonstrate that low income adversely contributed to asthma exacerbations and asthma treatment failure.^13^^,^^21^ Cardet et al^13^ reported that the rate of asthma treatment failure and asthma exacerbations was significantly higher among those with a household income <$40,000, compared with those with a household income ≥$50,000.

Along with individual-level SES, studies examined the impact of several neighborhood-level socioeconomic characteristics, such as the proportions of poverty, educational attainment, and health literacy at the neighborhood level. A ratio of ED visits and hospitalizations relative to outpatient visits for asthma increased if children lived in low-income neighborhoods with high poverty rates in LAC.^11^ The combination of low income and high poverty rate was identified as a determinant of asthma incidence. Zanobetti et al^12^ found the increased incidence among children living in neighborhoods with higher rates of low-income households and poverty after adjusting for individual-level characteristics. In contrast to our expectation, an increase in percentage of African Americans was associated with decreased ED visits for asthma. Such a negative association may be explained by an ecological fallacy or recent outmigration of African American residents in LAC. Consistent with our results, Southern California Association of Governments reported that African American population declined by >10% between 2006 and 2002.^22^ It may also suggest that environmental risk factors (eg, traffic emission) play more dominant roles in asthma outcomes than social or structural factors. This unexpected finding warrants further investigation in future studies.

The literature demonstrates no substantial findings to explain a mechanism by which neighborhood SES contributes to asthma outcomes. Zárate et al^5^ documented the limitations of neighborhood SES to explain the spatial distribution of asthma ED visits in Central Texas, accentuating the latent effects of unobserved characteristics. To address this gap, studies suggest that the link between neighborhood socioeconomic factors and asthma should be explained by multiple or complex pathways rather than a single, explicit risk factor, particularly in disadvantaged neighborhoods. Asthma incidence and exacerbation may arise from multidimensional interactions among various social and structural determinants, such as education, poverty, racial composition, access to health care, residential segregation, and discrimination.^1^^,^^2^^,^^12^^,^^23^

As a strength, this study employed the change score approach to assess the short-term temporal changes in the neighborhood characteristics. We argue that the results of this approach strengthened the basis for drawing causal inferences between neighborhood characteristics and ED visits and lowered the likelihood of spurious causality relative to cross-sectional designs. Therefore, the findings of this study provide insight into how to respond to the increased burden of asthma through more efficient allocation of local resources. This study also used detailed neighborhood-level data and investigated local characteristics by categorizing them into the 3 domains, including demographic, socioeconomic, and environmental domains. LAC has racially, culturally, and socioeconomically diverse communities. The neighborhood level data of LAC should be among the most representative resources to examine how neighborhood characteristics contribute to asthma outcomes. LAC shows a distinct pattern of racial/ethnic residential segregation. For example, South Los Angeles has a relatively higher proportion of Hispanic and African American populations. Such segregation may confound or moderate the relationship between socioeconomic characteristics and asthma outcomes, potentially leading to misleading results. Consequently, the findings of this study should be validated by future studies that use racially more homogeneous, neighborhood-level data from other metropolitan areas.

As an ecological study, this study did not account for biological, behavioral, and socioeconomic characteristics at the individual level. In addition to neighborhood determinants, asthma outcomes are often associated with those individual characteristics, such as genetics, comorbidities, educational attainment, health behavior, access to health care, and adherence to the regimen.^24^ They are also linked with household level risk factors, such as residential distance to roadways and poor housing conditions (eg, indoor pollutants, congestion, and poor maintenance).^4^^,^^11^^,^^17^ Hence, we suggest the use of hierarchical and longitudinal study designs that incorporate individual-, household-, and neighborhood-level characteristics across multiple time points to better disentangle these multilevel impacts on asthma outcomes.

### Conclusion

We found that an increase in PM_2.5_ was associated with increased ED visits for asthma at the neighborhood level, supporting the role of PM_2.5_ as among the fundamental environmental risk factors for asthma. Among the neighborhood socioeconomic characteristics, an increase in MHI was associated with decreased ED visits. However, we found little support for the beneficial impact of improved neighborhood SES. From a public policy standpoint, our findings underscore that, in the short term, reducing traffic emissions may more effectively lower the burden of asthma-related health care use than improving the neighborhood’s SES, which changes more slowly over time.

Policy interventions have been implemented through collaboration among local stakeholders, including air quality management districts, environmental protection agencies, regional planning organizations, and public health departments in Southern California. Federal- and state-level investment may help reduce the use of heavy-duty vehicles. To further decrease traffic emissions, residents should be encouraged to use public transit, adopt flexible hours, and switch to clean vehicles or fuels, while ongoing efforts to improve socioeconomic conditions in low-income neighborhoods should continue.

## Disclosure statement

Disclosure of potential conflict of interest: The authors declare that they have no relevant conflicts of interest.

Data sharing statement: Due to the nature of the research, supporting data are not available.
